# Development and genetics in the evolution of land plant body plans

**DOI:** 10.1098/rstb.2015.0490

**Published:** 2017-02-05

**Authors:** C. Jill Harrison

**Affiliations:** School of Biological Sciences, University of Bristol, 24 Tyndall Avenue, Bristol BS8 1TQ, UK

**Keywords:** land plant, evolution, evo-devo

## Abstract

The colonization of land by plants shaped the terrestrial biosphere, the geosphere and global climates. The nature of morphological and molecular innovation driving land plant evolution has been an enigma for over 200 years. Recent phylogenetic and palaeobotanical advances jointly demonstrate that land plants evolved from freshwater algae and pinpoint key morphological innovations in plant evolution. In the haploid gametophyte phase of the plant life cycle, these include the innovation of mulitcellular forms with apical growth and multiple growth axes. In the diploid phase of the life cycle, multicellular axial sporophytes were an early innovation priming subsequent diversification of indeterminate branched forms with leaves and roots. Reverse and forward genetic approaches in newly emerging model systems are starting to identify the genetic basis of such innovations. The data place plant evo-devo research at the cusp of discovering the developmental and genetic changes driving the radiation of land plant body plans.

This article is part of the themed issue ‘Evo-devo in the genomics era, and the origins of morphological diversity’.

## 1. Introduction

**Figure 1.**
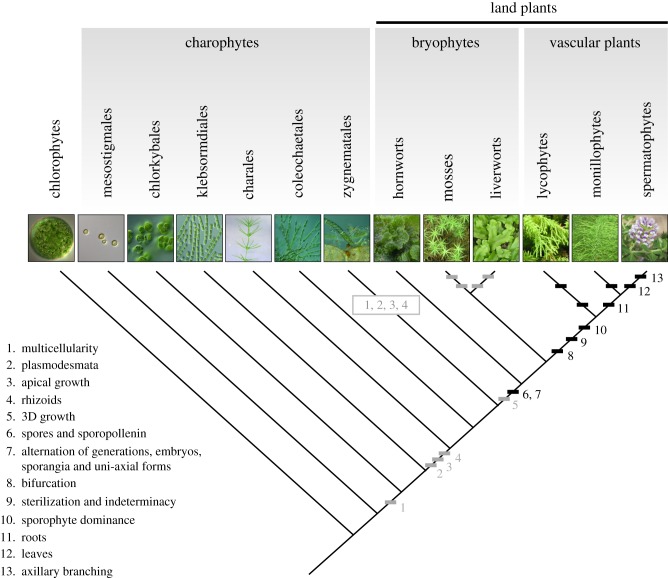
Gametophytic (grey bars) and sporophytic (black bars) innovations in the radiation of plant body plans. The earliest plant forms were unicellular freshwater algae, and land plants emerged from a grade of charophyte algae [4,5]. Multicellularity (1), plasmodesmatal cell to cell connections (2), specialized apical cell fates (3) and rhizoids (4) were evolutionary innovations preceding the origin of land plants, and 3D apical growth (5) evolved concomitantly with land plants [5–9]. Spores and dessication-resistant spore coats (6) are thought to have evolved prior to multicellular sporophytes (7). Although the earliest sporophyte forms were uni-axial terminating in sporangium formation, subsequent forms bifurcated (8) and this innovation preceded the origin of indeterminate axial forms (9) in vascular plants. A shift to sporophyte life cycle stage-dominance (10) emerged with vascular plants, and roots (11) and leaves (12) evolved independently in vascular plant lineages [9]. An axillary branching pattern evolved in spermatophyte precursors [10] and in liverwort and moss gametophytes [11,12]. Photos from left to right: *Erymosphaera* sp., *Mesostigma viride*, *Chlorokybus atmophyticus*, *Klebsormidium flaccidum*, *Chara braunii*, *Coleochaete pulvinata*, *Spirogyra* sp. kindly provided by Chuck Delwiche. Photo of *Folioceros glandulosus* kindly provided by Li Zhang. Photos of *Polytrichum commune*, *Marchantia polymorpha*, *Huperzia phlegmaria*, *Equisetum hymale* and *Ortholobium frutescens* by Jill Harrison (not to scale).

Land plants (embryophytes) originated around 470 million years ago among a crust-forming terrestrial microbiome of bacteria, cyanobacteria, algae, lichens and fungi [1–3]. Land plants emerged from the charophyte lineage, and charophyte algae have unicellular ancestral forms and life cycles in which meiosis immediately follows zygote formation (figure 1) [4,5,13]. Through time, there was a general trend towards the evolution of more complex multicellular algal forms with specialized cell and tissue types but no further elaboration of the diploid life cycle stage (figure 1) [5,6]. This pattern of life cycle progression was superseded by life cycles with alternating mulitcellular haploid (the gametophyte) and diploid phases (the sporophyte) in land plants [7]. The relative dominance of each phase shifted from the gametophyte (as in bryophytes) to the sporophyte (as in vascular plants) during evolution, and land plant forms have diversified following independent trajectories in each life cycle stage [7,14]. The major extant lineages of land plants were established by *ca* 360 million years ago including hornworts, liverworts, mosses, lycophytes, monilophytes and spermatophytes (figure 1) [15,16]. The evolution of these groups drove soil formation, increased primary productivity, and impacted on weathering and global climates [10,16,17].

The distinct morphologies of each land plant group reflect their use of divergent developmental and genetic programmes in generating form [18]. The basic building blocks of plant form typically include shoots, branches, leaves and roots whose relative arrangement and growth generate diversity [19]. The absence of these organ systems in fossil and living relatives of the earliest vascular plants and their progenitors has led to alternative interpretations of modular growth and left open questions about the nature of transitions in form occurring during evolution [8,15,20–25]. Many of the most ancient plant groups have a sparse fossil record [13,16], so we can only infer the sequence and nature of change underpinning the radiation of plant forms by comparing the characteristics of their living descendants. Phylogeny and evo-devo approaches to the evolution of plant form are starting to illuminate the nature of developmental and genetic change driving the radiation of form [15,18,21–25]. This review aims to give an overview of recent developmental and genetic findings relating to innovations driving the evolution of land plant form in a contemporary phylogenetic framework.

## 2. Contemporary views of plant phylogeny

**Figure 2.**
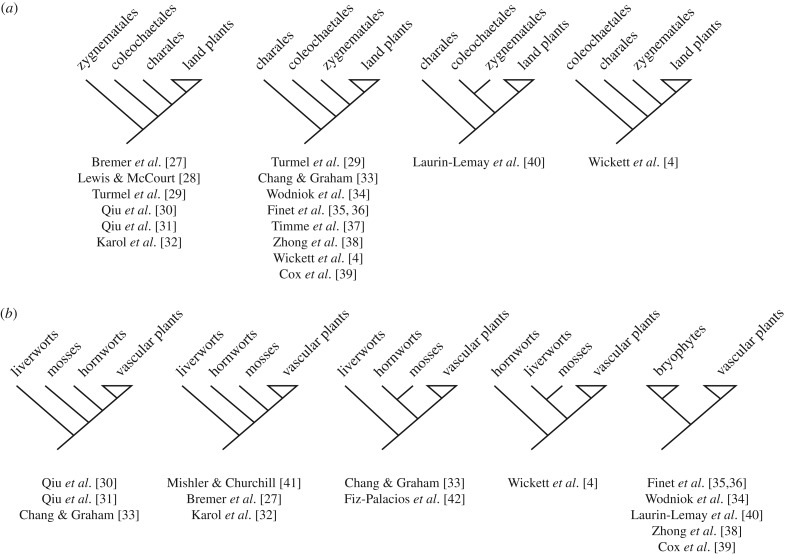
Currently supported sister group relationships between (*a*) charophyte algae and land plants and (*b*) bryophytes and vascular plants.

Comparison of plant form in a phylogenetic framework provides rigorous testable hypotheses of evolutionary change. Over the last 30 years, phylogenetic approaches have shifted from using morphological datasets to single-gene molecular datasets and later to multigene and genomic datasets, and reconstruction methods have shifted from favouring parsimony to likelihood [4,26]. Hypotheses of relationship within streptophyte algae and basal land plant lineages have been particularly labile given these changing methodologies (figure 2) [4]. Three lineages of charophyte algae are postulated to have a close relationship to the monophyletic land plant clade: Charales, Coleochatales and Zygnematales [4,27–40]. The four currently supported hypotheses of sister relationship to land plants are shown in figure 2*a*. Within the land plants bryophytes are basal, but among bryophytes there are five currently supported hypotheses of sister relationship to the monphyletic vascular plant group (figure 2*b*) [4,27,30–36,38–44]. These alternative arrangements bear on interpretation of the direction of change in morphological evolution, but do not preclude identification of the key characteristics contributing to the radiation of land plant forms. While many of the innovations contributing to land plant evolution were metabolic or physiological [22,45,46], this review specifically focuses on innovations contributing to the evolution of plant form (figure 1).

## 3. Innovations prior to the colonization of land

### (a) Multicellularity, filaments and apical growth

The first innovations occurred in charophyte algae prior to the colonization of land. Whereas charophyte forms are varied including unicells (e.g. *Mesostogma viride*), unbranched filaments with no apical growth (e.g. *Klebsormidium flaccidum*), branching filaments with apical growth (e.g. *Coleochaete pulvinata*) and thallose forms (figure 1) [5], bryophyte gametophytes have branching filaments with apical growth (e.g. *Physcomitrella patens*) or are thallose (e.g. *Folioceros glandulosus*, *Marchantia polymorpha*). Thus, elements of morphology were maintained when plants colonized land. Transitions between ancient unicellular and multicellular forms can be driven in both directions in the laboratory. In the unicellular chlorophyte alga *Chlamydomonas reinhartii (Chlamydomonas)*, heterologous expression of the retinoblastoma cell cycle regulator from the colonial alga *Gonium pectorale* can induce the formation of colonial morphs [47]. Experimental evolution experiments can drive the acquisition of multicellular form when a selection pressure for settling (sedimentation) is imposed, again pointing to small genetic changes in the evolution of multicellularity [48]. Reverse genetic approaches in the moss *Physcomitrella patens (Physcomitrella)* can drive morphology in the other direction. The protein prenylation-defective *ggb* and *plp* mutants are unable to attain the usual filamentous form. Instead they have small, round aggregated cells that lack polarized growth, oriented division and apical growth, and mutants resemble some charophytes [49,50]. Genome sequence of the freshwater alga *Klebsormidium flaccidum* is similar to land plant sequences and supports the notion that the genetic distance between charophyte algae and land plants is small [51]. These data demonstrate that transitions between unicellular, clustered and filamentous forms with apical growth can involve small genetic changes in both green algae and bryophytes, and they point to potentiating developmental changes for the radiation of land plant forms in their algal ancestors.

## 4. Innovations in the transition to land

### (a) Three-dimensional apical growth

Plant cells are bounded by a cell wall, so overall plant form reflects cell division plane orientation and growth during development. While algae are typically constrained to filamentous or mat-like planar (two-dimensional (2D)) forms, land plant forms can initiate (three-dimensional (3D)) growth in multiple axes (figure 1), and this evolutionary switch arose by the innovation of rotating division plane orientations in stem cells in plants' growing tips [8]. The evolutionary transition from 2D to 3D growth is recapitulated during the normal development of modern moss gametophytes, which undergo a filamentous growth phase (like algae) prior to the onset of 3D leafy shoot growth [52]. During the 2D growth phase, stem cells at the primary filament tip elongate by tip growth [53]. New growth axes initiate as foci of tip growth in sub-apical cells, and the new cells formed can either become secondary filaments (95%) or leafy shoots (5%) [54]. These differences in fate are sometimes evident at the single-celled stage due to a cylindrical (2D fate) or swollen (3D fate) cell appearance [52,54]. APB transcription factors belonging to the AP2 family promote the acquisition of 3D fate, and are necessary for leafy shoot formation. *APB* genes are expressed in primary filaments and new secondary outgrowths. If *APB* expression is lost in a secondary outgrowth, the new cell goes on to form a secondary filament, undergoing divisions perpendicular to the main growth axis. If *APB* expression is retained, the newly formed cell swells and undergoes an oblique division, marking the onset of 3D growth [54]. Although APBs seem to act as a molecular switch to specify 3D fate, the mechanisms regulating stem cell division planes at the onset of 3D growth are unknown. The calpain protease DEK1 orients cell division planes slightly after the onset of 3D growth, and DEK1 feeds back onto the 3D growth initiation process [55–57]. A transcriptomic analysis of 3D growth induction in *Physcomitrella* identified homologues of genes that regulate asymmetric cell division and shoot patterning in flowering plants [58], and downstream regulators include *PpCESA5*, a cellulose synthase [59]. These results mean that we are now poised to solve the problem of how 3D growth arises in *Physcomitrella* gametophytes, whose development exemplifies the 2D to 3D growth transition occurring in land plant evolution.

### (b) Spores, sporopollenin and sporangia: meiotic changes preceding sporophyte evolution

The innovations above occurred prior to or during the colonization of land in plants with gametophyte phase-dominant life cycles. The nature of morphological and developmental transition driving the evolution of multicellular sporophyte forms is not yet clear due to a thin fossil record. The earliest evidence of land plants comes from fossilized spore monads, dyads and tetrads that date back *ca* 470 million years [2,60]. The affinity of such ‘cryptospores’ is uncertain, but their wall structure is similar to the layered wall structures of some embryophyte fossil spores and living liverwort spores [2,60]. The earliest land plant macrofossils comprise sporangial fragments dating back around 450 million years [61].

The desiccation-resistant sporopollenin-coated spores that characterize land plants are thought to have evolved prior to the evolution of multicellular sporophytes [62]. Sporophytic multicellularity is proposed to have arisen by the interpolation of mitotic divisions into the meiotic developmental programme, and variation in fossil spore aggregation patterns and morphology suggests an early phase of evolutionary change in the timing of meiotic cell division relative to sporopollenin deposition [60,63–65]. Variation in spore form is sparsely reflected in living bryophytes, but the liverworts *Haplomitrium gibbsiae* and *Sphaerocarpus michelii* have permanent spore dyads and tetrads, respectively, and may represent a relictual state [66,67]. Sporopollenin production pathways are partially conserved within the land plants, and recent reverse genetic studies have determined that sporopollenin is required for spore viability [68–71].

### (c) Alternating gametophyte and sporophyte generations

Genetic evidence suggests that early variability in meiotic division pathways and multicellularity in the earliest sporophytes could have been linked. In the unicellular chlorophyte alga, *Chlamydomonas reihardtii*, plus and minus mating identities are conferred by the TALE class homeodomain transcription factors GSP1 (a BELL protein) and GSM1 (a KNOX protein), respectively [72]. After mating, protein heterodimers form to activate zygote development, and ectopic co-expression of *GSM1* and *GSP1* is sufficient to trigger zygotic gene expression and meiosis [72]. BELL and KNOX proteins were inherited by land plants and their ancestral role in life cycle progression is preserved in bryophytes [73,74]. Knockouts and over-expressors in *Physcomitrella* show that *PpBELL1* activity is necessary and sufficient for sporophyte development [74]. A *KNOX* duplication occurred prior to the colonization of land giving rise to *KNOX1* and *KNOX2* classes [75,76]; the *KNOX1* gene *MKN2* regulates sporophyte development [77,78] and *KNOX2* genes suppress filament development in sporophytes [73]. PpBELL1 can heterodimerize with all five moss KNOX proteins, and heterodimerization with MKN2 may be responsible for the activation of sporophyte development when *PpBELL1* is ectopically expressed [74]. *PpBELL1* and *MKN2* act downstream of the POLYCOMB REPRESSIVE COMPLEX 2 (PRC2) components PpFIE and PpCLF, and *Ppfie* and *Ppclf* mutants activate aspects of development characteristic of sporophytes in the gametophyte generation [74,79,80]. *KNOX1* and *KNOX2* genes also affect sporangium development in *Physcomitrella*. In combination, data implicate *KNOX* and *BELL* genes in life cycle progression, meiosis and multicellular development at either side of the transition to land.

Further genetic mechanisms involved in the appearance of multicellularity and 3D growth in sporophytes are largely unknown because perturbations to bryophyte gametophyte development impair fertility, so separate mutants must be generated in each life cycle stage to study the function of the same genes. The *Physcomitrella* transcription factors PpLFY1/PpLFY2 and PpWOX13LA/PpWOX13LB are necessary for multicellular development [81,82] and polar auxin transport by PIN auxin transporters also regulates fertility and sporophyte development [83,84]. Recent progress has identified sporophyte-specific promoters that could be used to generate conditional mutants to dissect the function of these regulators of sporophyte development [74,85].

### (d) Uni-axial forms

**Figure 3.**
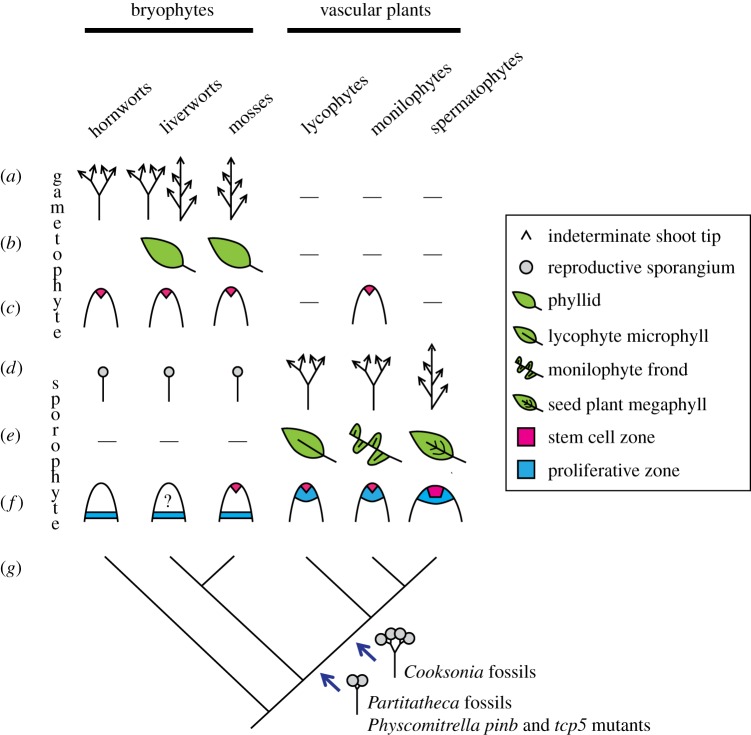
Key steps in the evolution of shoot form [11,25,60]. (*a*) Bryophyte gametophytes have diversely branching indeterminate shoots that may or may not iterate gametophytic leaves (*b*) and arise from a single apical stem cell (*c*) [11]. By contrast, bryophyte sporophytes have uni-axial forms that terminate in sporangium formation (*d*). The mechanisms for axis establishment vary between bryophyte groups (*f*). While hornwort axes extend by intercalary proliferation, the mechanism in liverworts may involve intercalary proliferation or just expansion. Mosses have apical and basal cells that establish the apical basal axis before the intercalary mersitem contributes to medial proliferation [11,65]. The origin of indeterminacy in vascular plants coincides with the displacement of sporangia away from shoot tips, and the juxtaposition of apical stem cell and proliferative zones. Fossil and mutant intermediary forms between bryophytes and vascular plants (*g*) suggest that a capacity for bifurcation arose before indeterminacy [60,83,85]. Leaves have five independent origins (*b*,*e*), two in bryophyte gametophytes and three in vascular plant sporophytes. For this reason each leaf type has a specific name [86,87].

While there is no fossil record of the earliest multicellular sporophyte forms, phylogeny suggests that uni-axial sporophytes terminating in sporangium formation are basal (figure 3*d*). Uni-axial forms are retained by living bryophytes, but the pattern and extent of development differs between bryophyte lineages, so it is not yet clear which pattern is ancestral. Although axial elongation in hornworts occurs from a ‘basal’ intercalary proliferative region that extends the apical basal axis and maintains spore production, liverwort sporophytes elongate principally by cell expansion (figure 3*f*) [11,65]. Mosses have transitory apical and basal cells that divide to form an embryonic axis, and an intercalary proliferative region in the middle of the axis later serves to elongate the stem and raise the sporangium out of the parent plant (figure 3*f*) [65,88]. Surgical decapitation experiments in mosses suppress axial elongation [89] and cytokinin can compensate for decapitation, suggesting that apically produced cytokinin promotes elongation. If applied to intact sporophytes, cytokinin can also induce extensive (sterile) axial elongation by prolonging activity of the intercalary proliferative region [89]. These data suggest that mosses have a latent capacity for proliferation that is normally suppressed by hormonal interplay between the apical cell, the intercalary zone and the sporangium.

## 5. Innovations in the origin of vascular plants

### (a) Bifurcation

While bryophyte morphology suggests that the most ancient sporophyte forms were uni-axial and terminated in sporangium formation, vascular plant forms are multi-axial and have shoot tips that proliferate indeterminately without terminating in sporangium formation (figure 3*d*). Morphological innovations at the bryophyte to vascular plant divergence are hard to unravel because the morphology of living bryophytes and vascular plants is disparate, and this disparity has led to much speculation about the nature of change [15,21–23,25,90–93]. Some of the earliest plant macrofossils share characteristics with bryophytes and vascular plants and point to developmental changes at this juncture (figure 3*g*) [60]. Their simple bifurcating forms terminate in sporangium formation, but inference of apical or proliferative activities during development has not yet been possible. The potential to use moss sporophytes as a ‘bottom up’ entry point to understanding the innovation of bifurcating forms is demonstrated by rare natural moss variants and mutants that bifurcate with axes terminating in sporangium formation [83,85,94]. Reverse genetic work in *Physcomitrella* has shown that perturbation of polar auxin transporter (PINB) or TCP transcription factor (TCP5) function can induce single or multiple bifurcations with each resultant axis terminating in sporangium formation, but the developmental basis of mutant phenotypes is not yet clear [83,85]. Modern lycophytes and monilophytes offer the possibility of ‘top down’ insights into mechanisms of bifurcation as their meristems have one to a few stem cells, a state thought to be ancestral within the vascular plants. A clonal analysis in the lycophyte *Selaginella kraussiana* showed that bifurcation involves amplification and segregation of stem cells in the shoot tips, and suppression of genetic pathways for proliferation [95–97]. Bifurcation in the fern *Osmunda regalis* is thought to involve stem cell division, and the development of fern genetic models opens the possibility of mechanistic insights [98–100].

### (b) Indeterminacy and the displacement of sporangium formation away from shoot tips

**Figure 4.**
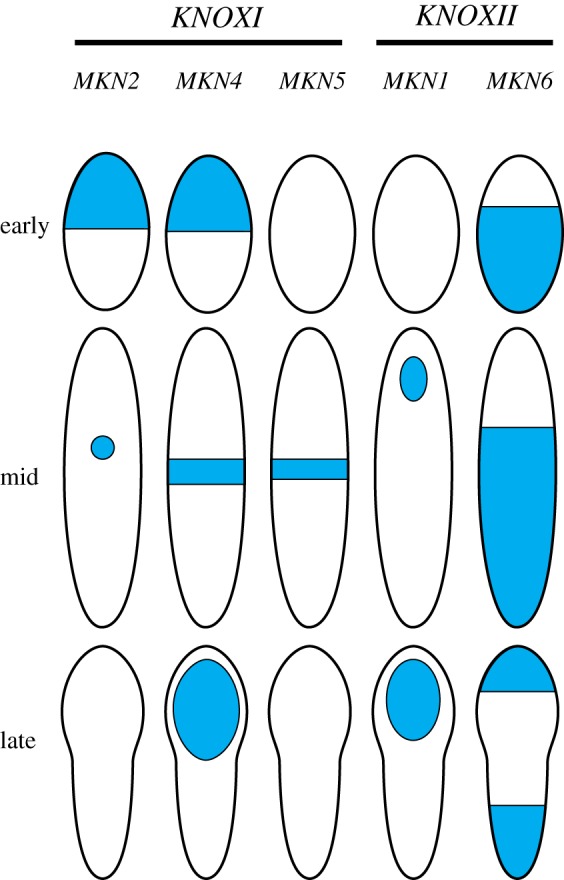
*KNOX1* and *KNOX2* expression patterns in *Physcomitrella patens* sporophytes redrawn from [73,78]. *KNOX* expression patterns in moss sporophytes are dynamic. During early embryo development, the *KNOX1* genes *MKN2* and *MKN4* are expressed in the apical half of the embryo including the apical cell, and expression then narrows to medial a spot (*MKN2*) or band (*MKN4, MKN5*) coinciding with the position of intercalary proliferation [78]. Later on *MKN4* is expressed the sporangium [78]. The *KNOX2* gene *MKN6* has a basal early embryonic expression pattern that is converse to *MKN2* and *MKN4* expression patterns [73]. Later on during development *MKN1* is expressed in the sporangium and *MKN6* is expressed in bands across the embryo [73]. These patterns are suggestive of roles for *KNOX* genes in mapping out developmental domains in determinate uni-axial sporophytes. In conjunction with converse mutant phenotypes, converse patterns of *KNOX1* and *KNOX2* expression suggest antagonistic functions. (Online version in colour.)

Although bifurcating forms with terminal sporangia are prevalent in early vascular plant fossils, forms with spatially distinct proliferative and reproductive activities are also represented, having lateral sporangia or sporangia on leaves [101,102]. This mix of characteristics suggests that the displacement of sporangial development pathways away from shoot tips was pre-requisite to the evolution of indeterminate meristem functions. There is some evidence from either side of the bryophyte to vascular plant divergence that *KNOX1* and *KNOX2* genes could have contributed to the evolution of indeterminacy and sterilization. *Physcomitrella KNOX1* genes are first expressed in the apical half of the embryo before expression narrows down to a spot subtending the sporangium (*MKN2*) or a band whose position coincides with the position of the intercalary proliferative region (*MKN4*/*5*; figure 4) [78]. MKN2 activity is necessary for axial elongation, perhaps by modulating intercalary proliferative activity [78]. Axial elongation following cytokinin application [89] and suppression of elongation in *mkn2* mutants [78] suggests a potential link between cytokinin and KNOX1 activities, which is significant given that a KNOX-cytokinin regulatory loop promotes indeterminacy in *Arabidopsis* [103,104], and expression analyses in a lycophyte show that *KNOX1* genes are conserved regulators of indeterminacy in vascular plants [95]. The possibility of roles for *KNOX* genes in sterilization is raised by *knox1* and *knox2* mutant phenotypes in *Physcomitrella*. *KNOX1* and *KNOX2* expression patterns are somewhat complementary in sporophytes (figure 4), and while the *KNOX1* gene *MKN2* promotes sporangium development, the *KNOX2* genes *mkn1* and *mkn6* are necessary for sporangium development [73,78]. *KNOX1* and *KNOX2* genes have antagonistic functions in other plants [105], and *KNOX1* and *KNOX2* genes may act antagonistically to regulate sporangium development in *Physcomitrella*. *MKN* function links proliferative and reproductive activities in moss sporophytes, and a key point for future research will be to understand how KNOX evolution may have teased these activities apart during the evolution of sporophytic indeterminate meristem functions.

## 6. Innovations in vascular plant diversification

### (a) Meristems

Morphological changes in the evolution of land plant forms correlate with changes in meristem function through time, and indeterminate meristems have evolved independently in bryophyte and fern gametophytes and vascular plant sporophytes (figure 3) [7]. While gametophyte meristems comprise a single apical stem cell, vascular plant meristems have one to many stem cells capping a more rapidly proliferative zone (figure 3*c*,*f*) [11,106–108]. The intercalary proliferative zones of bryophyte sporophytes may be homologous to the proliferative zones of vascular plant meristems, but the mechanisms by which apical stem cell activity originated in sporophytes are unknown. The juxtaposition of stem cell and proliferative zones may have preceded the origin of indeterminacy in vascular plants (figure 3*f*). Molecular work suggests that there has been no large-scale co-option of genes regulating meristem function from the gametophyte to the sporophyte stage of the life cycle [78,82,109], and transcriptomic work suggests that vascular plant meristems may have evolved independently in lycophytes, monilophytes and spermatophytes [25,110]. *PIN* genes are an exception and PIN-mediated auxin transport drives meristem function in both life cycle stages in a moss [83].

### (b) Leaves

A primary function of indeterminate meristems is to iterate leaves in regular patterns around the stem to optimize light interception during photosynthesis [19]. Phylogeny shows that leaves had at least five independent origins, evolving in liverwort and moss gametophytes, and also in lycophyte, monilophyte and seed plant sporophytes (figure 3*b*,*e*) [86]. Steps in the evolution of vascular plant leaves are represented in the fossil record, and leafless precursors in each lineage point to non-homology of sporophytic leaf types, a subject that is well reviewed elsewhere [86,111]. Non-homology of leaf types is also supported by widely divergent patterns of leaf development in each group. Liverwort and moss phyllids develop from a single cell, and with the exception of the midvein in mosses, comprise a single cell layer [11]. Lycophyte microphylls can develop from two juxtaposed epidermal cells, with inner tissue layers established by division of the adaxial epidermis after medio-lateral and proximo-distal axes of symmetry are established [96]. Monilophyte fronds have a shoot-like nature, developing from a single apical cell [112], and seed plant leaves develop from a pool of cells recruited from the flanks of the multicellular shoot apical meristem [106]. Axes of leaf symmetry are largely inherited from the parent meristem [19].

In line with these divergent leaf morphologies and patterns of development, transcriptomic analyses have identified largely divergent genetic pathways for leaf development in each vascular plant lineage [110]. However, reverse genetic studies have identified three pathways that have been co-opted multiple times to regulate leaf development during evolution. PIN-mediated auxin transport is a key determinant of leaf initiation patterns and development in flowering plants, and PIN-mediated auxin transport was independently co-opted to regulate phyllid initiation and outgrowth in moss and probably also lycophyte microphylls [83,113,114]. ARP transcription factors are a second key driver of leaf initiation and development in flowering plants, and their action in downregulating *KNOX* expression was independently co-opted to regulate lycophyte microphyll development [95,115]. HD-zip transcription factors regulate leaf polarity and vascular development in flowering plants, and *HD-zip* genes have been independently recruited to regulate different aspects of leaf development in mosses and lycophytes [109,116–118]. Each of these gene families has undergone lineage-specific duplications during evolution, and the genetic networks regulating leaf development are likely to be lineage-specific [95,117–119].

### (c) Axillary branching

Shooting forms in land plants are further characterized by extensive branching, which confers fitness advantages allowing increase in size, plastic growth responses, persistence over long time frames and amplification of reproductive pathways [120]. While bifurcation is likely to be the ancestral branching pattern in gametophytes and sporophytes, axillary branching arose independently in moss and liverwort gametophytes and seed plant sporophytes (figure 3*a*,*d*,*g*) [121]. Most of our understanding of branching has been gained from studies in flowering plants in which branches initiate as a result of a drop in the levels of auxin and a rise in the levels of cytokinin in cells at the base of leaf primordia [122,123]. Cells that attain branch fate in this manner can activate branch outgrowth in response to hormonal cues integrated across the plant later in development [121,124]. Shoot apices and young leaves play a major role in regulating branch outgrowth patterns as they produce auxin that is then transported away from the shoot tips via the polar auxin transport stream to suppress branching, and cytokinin antagonizes the action of auxin [124]. Strigolactone is a third hormonal regulator of branching, inhibiting or promoting branch outgrowth depending on the auxin transport status of the plant [125–127]. The mechanisms driving the evolution of lateral branching are largely unknown. However, lateral branches in *Physcomitrella* gametophytes arise by respecification of epidermal cells in leaf axils to apical cell fate [12]. Although this process is modulated by an interplay between auxin, cytokinin and strigolactone, the auxin transport route is non-polar and likely to involve plasmodesmata, and branch activation is likely to directly reflect downstream outputs of hormone signalling [12].

## 7. Underground innovations

### (a) Rhizoid-based rooting systems

The earliest rooting systems originated prior to the colonization of land and were rhizoid based, comprising unicellular or multicellular projections that function in anchorage and mineral uptake [128,129]. The genetic mechanisms regulating rhizoid development predate the origin of land and are conserved with mechanisms regulating root hair development in flowering plants [130,131]. *RSL* Group VIII bHLH transcription factors are positive regulators of rhizoid development that underwent an early duplication to form Class 1 and Class 2 clades [130,132,133]. Each class is represented by a single gene in the liverwort, *Marchantia polymorpha*, and loss- and gain-of-function mutations in *RSL1* have revealed that it is necessary for and promotes the formation of all epidermal cellular projections, a role that is conserved with mosses [130]. *RSL* copy numbers in both classes have been maintained at low levels through to a total of 7 in mosses to 8 in lycophytes and a maximum of 10 in flowering plants [134]. *LRL* Group XI bHLH transcription factors amplified from a base number of 1 in charophytes via two vascular plant-specific duplications to form 3 classes through time [131]. While liverwort and moss *LRL*s promote rhizoid development, *Arabidopsis* *LRL*s can either promote (Class I) or repress (Class II) root hair development, and both *RSL* and *LRL* regulatory networks have increased in complexity during plant evolution [131,134–136]. These networks have been repeatedly deployed to allow rhizoid or root hair development on different parts of plants during evolution indicating deep homology; the use of conserved genetic networks in the development of non-homologous structures [137].

### (b) Roots

Although vascular plants from the fossilized Rhynie Chert assemblage had rhizoid-bearing subterranean stems that performed a rooting function, true rooting systems diversified after shoot systems [138]. The features that distinguish roots from earlier axial forms are growth from a meristem with a root cap and gravitropism, and roots had independent origins in lycophytes and euphyllophytes (monilophytes and spermatophytes) or each euphyllophyte lineage [138]. Some of the earliest root systems fossilized from the ancient lycophyte forests that formed coal. These comprised bifurcating shoot-like axes (rhizomorphs) that initiated bifurcating rootlets in a spiral phyllotaxis and had root hairs [139,140]. Roots in living lycophytes comprise a system of bifurcating axes with hairs that either originate laterally during embryogenesis or initiate from modified aerial axes (rhizophores) during post-embryonic growth [138–141]. The developmental affinity of lycophyte rhizophores to roots or shoots is a long-standing debate in which the most recent evidence from shared protein abundance and *KNOX* gene expression points to shoot-like affinity [141–143]. The earliest euphyllophyte roots fossilized from extinct cladoxylopsid plants that resemble tree ferns, and their root systems comprised bifurcating axes initiating from the swollen stem base [129,144,145]. In living ferns, roots initiate either basally during embryogenesis or post-embryonically from the stem base, stems or rhizomes; lateral roots initiate from an endodermal cell layer [141,146]. The progymnosperm fossil relatives of seed plants had shrub or tree form with woody tissues and bifurcating (aneurophytes) or laterally branching roots (archaeopterids) as in living seed plants [147,148].

Trends in root architecture evolution somewhat mirror trends in the evolution of shoot architecture, and euphyllophyte rooting systems are hypothesized to have a shoot-like origin [7,149,150]. Although there is a good understanding of the molecular mechanisms regulating root architecture in flowering plant models [151], knowledge transfer to identify the mechanisms underpinning root evolution is limited. The homology of lcyophyte, monilophyte and spermatophyte roots is supported by an analysis of *WOX* gene function showing that root stem cell expression is conserved [152]. The distinct origin of lycophyte and monilophyte and spermatophyte rooting systems is supported by a phylogenetic analysis showing that lycophytes lack the *AS2/LOB* domain genes that act downstream of auxin to regulate root development in monilophytes and spermatophytes [153].

## 8. Themes

### (a) Gene duplication and antagonistic functions

Genomic approaches to plant evolution have shown that most of the gene families with important roles in generating flowering plant form are conserved to algae, and that gene copy numbers have amplified through time [51,154]. This amplification provides a source of genetic diversity, and has led to long-standing ideas about the contribution of gene duplication and diversification in function to the radiation of diverse forms [155,156]. A recurring theme to emerge from more recent studies in basal land plant lineages such as liverworts and mosses is that antagonistic gene functions arise following duplication. For instance, through time the ancient *KNOX* to *KNOX1* and *KNOX2* duplication lead to antagonistic functions for these gene classes in leaf development [105], and *bHLH* and *HD-ZipIII* duplications have given rise to antagonistic functions in root hair development and axillary meristem development, respectively [131,157]. The mechanisms by which such antagonistic transcription factor functions emerge are not yet clear but are accessible to experimental interrogation.

### (b) An upward outlook for the genetics of plant form

A second theme from recent plant evo-devo approaches is that models with low genetic redundancy but conserved gene families are bringing new findings that are broadly relevant across the plant tree of life. For instance, *Marchantia rsl* mutants have not only a rhizoidless phenotype but also defects in the initiation of other epidermal projections such as glandular hairs, which are ubiquitous in land plants [130]. This discovery lead to the identification of conserved roles for *RSL*s in regulating epidermal cell outgrowth in *Physcomitrella*, and may be taken further up the plant tree of life into vascular plants in the future [130]. Unpublished data from other laboratories are pointing in the same direction and are identifying new roles for conserved gene families that can be taken up the plant tree of life. These data demonstrate the potential of forward genetic approaches in bryophytes for gene discovery in gene regulatory network and signalling pathway analysis. They are relevant in the light of knowledge transfer to flowering plants where redundancy has previously masked gene function, and application of new knowledge to modify crop form may improve yields in future work.

### (c) Small genetic changes for major innovations

A third theme to emerge from developmental and genetic studies spanning the base of the plant tree of life is that single-gene mutations can induce discrete changes relevant to major evolutionary innovation [49,56,72,83]. While the morphological distance at the alga to land plant and bryophyte to vascular plant divergence points is wide, new fossils and mutants have started to generate intermediate forms [49,83]. In some instances, such forms can be interpreted in the light of stem and crown group morphologies to suggest stepwise body plan changes. In other instances the forms generated are clearly maladaptive, and potential transitions in form remain elusive [55,56]. Nevertheless, the advent of forward genetic approaches that have gone from phenotype to genotype in *Marchantia* and *Physcomitrella* brings opportunity for significant and imminent advances in testing the limits of plant forms in early diverging land plant lineages [130,158]. Mutant phenotypes arising are likely to be informative about the nature of morphological transition occurring in plant body plan evolution during the colonization of land, and genotyping will identify key genes for architectural change.
